# Distribution Patterns of *tfd*_I_ and *tfd*_II_ Gene Clusters and New Insights into the Formation of the Architecture of pJP4, a Canonical 2,4-dichlorophenoxyacetic Acid (2,4-D) Degradation Plasmid

**DOI:** 10.3390/ijms252010998

**Published:** 2024-10-12

**Authors:** Timur Iasakov

**Affiliations:** Ufa Institute of Biology, Ufa Federal Research Centre, Russian Academy of Sciences, Prospekt Oktyabrya 69, 450054 Ufa, Russia; iasakovtimur@gmail.com or iasakov@anrb.ru

**Keywords:** pJP4, plasmid, IncP-1, *tfd*, cluster, gene, 2,4-dichlorophenoxyacetic acid (2,4-D), distribution patterns, phylogenetic analysis, comparative genomics

## Abstract

Currently, pJP4 is one of the best-known plasmids for the biodegradation of xenobiotics that mediate the degradation of 2,4-dichlorophenoxyacetic acid (2,4-D), which is associated with serious health and environmental risks. Although the sequencing and proposed theory of pJP4 formation occurred almost 20 years ago (2004), pJP4 is still the model object of many studies focused on the biodegradation of 2,4-D. The uniqueness of this plasmid is due to the presence of two evolutionarily distinct gene clusters, *tfd*_I_ and *tfd*_II_, controlling the degradation of 2,4-D. Recent advances in plasmid biology, especially those concerning the characterization of new IncP-1 plasmids and the systematization of *tfd* gene cluster findings, serve as a basis for proposing new insights into the formation of the clusters’ architecture of the canonical plasmid, pJP4, and their distribution among other plasmids. In the present work, a comparative genomic and phylogenetic in silico study of plasmids with *tfd*_I_ and *tfd*_II_ clusters was carried out. The possible initial distribution patterns of *tfd*_I_ clusters among plasmids of different incompatibility groups (non-IncP-1) and *tfd*_II_ clusters among IncP-1 plasmids using the IS*1071*-based composite transposon were revealed. A new theory on the formation of the architecture of the *tfd*_I_ and *tfd*_II_ clusters of pJP4 through sequential internal rearrangements, recombination, and IS*JP4* insertion, is proposed. In addition, small gene clusters resulting from internal rearrangements of pJP4 (*tfd*_II_*SA* and ORF31/32) served as fingerprints for exploring the distribution of *tfd*_I_ and *tfd*_II_ clusters. The revealed patterns and formulated theory extend the frontiers of plasmid biology and will be beneficial for understanding the role of plasmids in bacterial adaptation to xenobiotic-contaminated environments.

## 1. Introduction

A 2,4-dichlorophenoxyacetic acid (2,4-D) degradation plasmid, pJP4, is a conjugative IncP-1 plasmid type with a length of about 88 kbp from *Cupriavidus pinatubonensis* JMP134 (initially identified as *Ralstonia eutropha*, *Alcaligenes eutrophus*, *Waustersia eutropha*, and *Cupriavidus necator*) [1,2,3,4]. Currently, the plasmid pJP4 is a unique model for xenobiotic degradation due to the combination of two 2,4-D degradation clusters (*tfd*_I_ and *tfd*_II_) [5,6,7]. The theory for the evolution of pJP4 plasmid and the formation of the two gene cluster architecture was proposed in 2004 by Trefault and colleagues [3]. According to them, a series of transpositions involving IS*1071*, with the *tfd*_I_ cluster on one side, and IS*JP4* with the *tfd*_II_ cluster on the other side, led to the formation of the original structure of this plasmid.

After, pJP4 plasmids containing *tfd* clusters were isolated from bacterial hosts distributed worldwide. Among them, there were both plasmids carrying a single [8,9] and combination [10,11] of *tfd* clusters. Recently, the diversity of *tfd* clusters has been classified through comparative genomic and phylogenetic approaches. The new classification of *tfd* gene clusters includes four types from different evolutionary lineages. At the same time, clusters of three types, namely *tfd*_I_, *tfd*_II_, and *tfd*_III_, have complete structures: *tfd*_I_*BFEDCT*, *tfd*_II_*AKBFECDR*, and *tfd*_III_*FAKBECDR*; in contrast to the fourth type, *tfd*_IV(A,B,C)_. This led to the reclassification of some *tfd* clusters of previously sequenced plasmids and the finding of their unusual architectures (e.g., *tfd*_I_*BFEDCT* and *tfd*_II_*SA*) [12].

The aim of this study is to reveal whether there are certain distribution patterns of *tfd*_I_ and *tfd*_II_ gene clusters among plasmids of different incompatibility groups. The study also aims to assess the relevance of the previous theory on the formation of the *tfd*_I_ and *tfd*_II_ clusters’ architecture of the canonical plasmid, pJP4, in light of new advances in plasmid biology. Here, an effort to resolve the above issues by employing comparative genomics and phylogenetics approaches is provided.

## 2. Results

### 2.1. Identification of Plasmids

**Figure 1:**
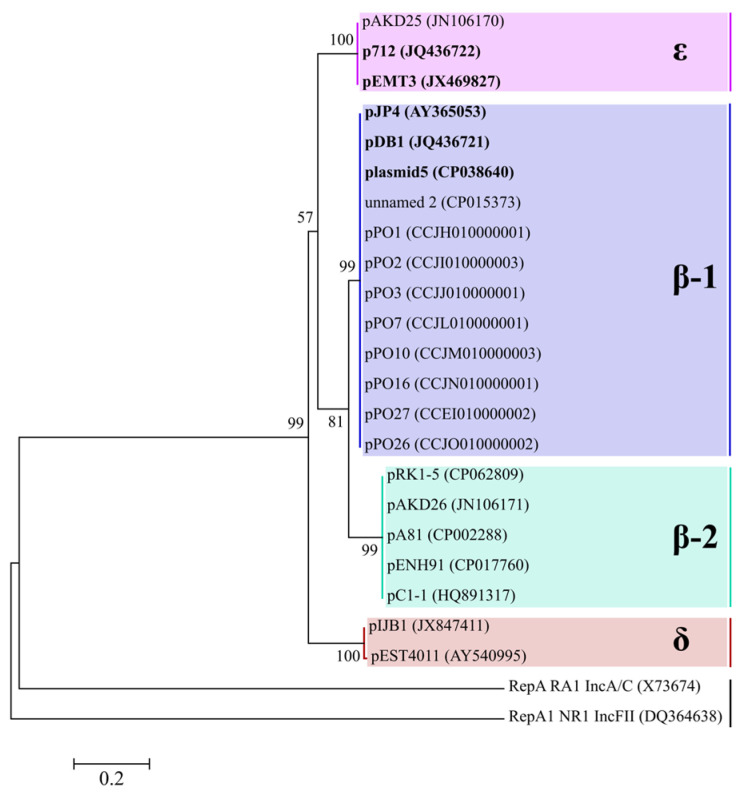
Maximum likelihood (ML) phylogenetic tree of replication initiation proteins TrfA of the IncP-1 plasmids. The IncP-1 plasmid subgroups are designated by Greek letters and highlighted with different colors. The proteins RepA and RepA1 of plasmids RA1 (IncA/C) and NR1 (IncFII), respectively, were used as an outgroup. Bootstrap support values higher than 50% are indicated at branching points. The trees are drawn to scale, with branch lengths measured in the number of amino acid substitutions per site. The scale bar at the bottom of the figure corresponds to 2 amino acid substitutions per 10 amino acids.

The majority of the plasmids containing *tfd* gene clusters were identified as members of the IncP-1 type according to their replicon typing, via a phylogenetic analysis of the deduced amino acid sequences of the replication initiation proteins encoded by the *trfA* gene and belonging to Rep_3 Superfamily (Table 1). In the ML analysis, TrfA proteins were recovered as monophyletic, strong-supported clades (Figure 1). The plasmids pJP4, plasmid5, and pDB1 clustered in a strong-supported β-1 clade, as well as p712 and pEMT3 in ε clade.

The similarity of the TrfA protein sequences of the IncP-1 plasmids varied from 77.9 to 100%. The plasmids with the *tfd*_II_ cluster, namely pJP4, plasmid5, pDB1, and the plasmids of the pPO line [11], had completely identical TrfA protein sequences, as did the plasmids p712 and pEMT3. Replication proteins of these two groups shared a similarity of 79.9%.

Plasmids pEMT1 and pkk4 were identified as members of IncHI2 and IncA/C incompatibility groups, respectively. Table 1 summarizes the comparative features of the plasmids used in the present study.

### 2.2. Comparative Genomics of the IncP-1 Plasmids Possessing tfd Type Gene Clusters

All plasmids analyzed had an architecture comprising a backbone and an accessory region. Each backbone had a modular structure, which included replication (*trfA* and *ssb* genes), conjugation (*tra* and *trb* genes), and maintenance (*kle*, *kor*, *klc*, and other genes) modules. The aligned nucleotide sequences of the plasmids had identities ranging from 69.2 to 98.1%. It should be noted that eight out of nine plasmids of the pPO line possessing *tfd*_I_ and *tfd*_II_ clusters (pPO1, pPO2, pPO3, pPO7, pPO10, pPO16, pP26, pPO27) were not completely sequenced [11] and, therefore, were not analyzed.

**Figure 2:**
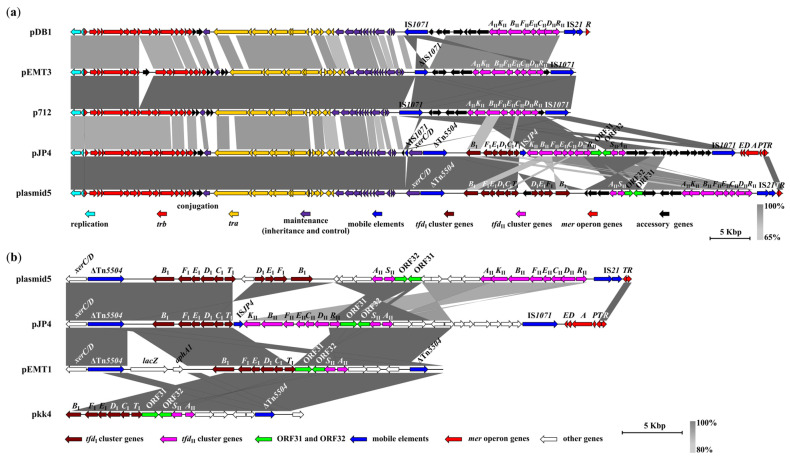
Comparative genomic analysis: (**a**) comparative genetic structures of the IncP-1 plasmids which contained *tfd* gene clusters; (**b**) comparison of *tfd*_I_-containing regions of IncP-1, IncHI2, and IncA/C plasmids. The plasmids and plasmid regions are shown by linear visualization with genes illustrated by labeled arrows, which are colored according to their functions (bottom). White and black unlabeled arrows indicate genes that are not discussed. The direction of the arrows indicates the location of the genes on the plasmid strands (right, forward strand; left, reverse strand). The truncated mobile elements are designated by delta symbol (Δ). The heat key (bottom right) illustrates the intensity of the grayscale-shaded regions according to blastn, which indicates the degree of identity between plasmids and plasmid regions. The scale in kilobase pairs (Kbp) is displayed on the bottom right side.

The identity levels of pJP4 plasmids and plasmid5 were 75.6%. Interestingly, they had an identical backbone structure, but some genes within the accessory regions showed a different or opposite arrangement on the plasmid (Figure 2a).

Plasmids pJP4, plasmid5, and pDB1, in contrast to plasmids p712 and pEMT3, carried genes of the *mer* operon, located at the end of the plasmids (Figure 2a). The three plasmids mentioned above had genes of *mer* operons as *merRTPADE*, *merRT*, and *merR*, respectively. Interestingly, a 27-nt sequence completely identical to the inverted repeat (IRL) of the mercury resistance Tn*501*-like transposon of the IncP-1 plasmids previously described [14], was identified at the 3′ end of the *merR* genes of pJP4 and pDB1, while it was absent in plasmid5.

The analyzed plasmids of the IncP-1 type contained several mobile elements, among which the IS*1071* insertion sequences (ISs) predominated (Figure 2a). Thus, accessory regions of pEMT3 plasmids and p712 were flanked by two copies of the IS*1071* in the same orientation, which in turn were flanked by short, direct repeats of 5’-aaatt-3’. At the same time, one of the flanking IS*1071* of pEMT3 was truncated.

The pJP4 plasmid possessed one complete copy of IS*1071*, oriented similarly to the pEMT3 and p712, and flanked to the accessory region from one of its ends. Additionally, a remnant of the IS*1071* was oriented in the opposite direction. Interestingly, the pDB1 plasmid possessed only one complete copy of IS*1071*, which was oriented similarly to the truncated copy of IS*1071* of the pJP4 plasmid.

Also, pDB1 and plasmid5 possessed an insertion sequence that belonged to the IS*21* family (92% identity at amino acid level with IS*Thsp19* transposase and 88% identity at amino acid level with IS*Pa63* helper protein) and that was adjacent to the genes of the *mer* operon.

Previously, Trefault and colleagues found a transposon in the pJP4 plasmid that was designated as Tn*5504* [3]. An analysis and reannotation of the pJP4 and plasmid5 revealed that: (i) the transposase gene of Tn*5504* currently has 84% identity at the amino acid level with IS*Psy30* and IS*Psy42* transposases (Tn*3* family), according to the ISFinder database; (ii) the open reading frame (ORF) from the 5′ end of the transposase gene previously suggested as encoding a resolvase of Tn*5504* according to the ISFinder database does not show significant levels of identity with previously known resolvases. Moreover, an annotation of the deduced amino acid sequence through the Conserved Domain Database (CDD) database allowed the identification of conserved domains of site-specific XerC and XerD recombinases. Thus, the putative transposon Tn*5504* is represented only by the transposase gene.

Among the plasmids analyzed, only pJP4 was the carrier of a single copy of the IS*JP4* insertion sequence belonging to IS*5* family.

### 2.3. Comparative Genomics of the IncP-1, IncA/C, and IncHI2 Plasmid Regions Possessing tfd_I_ Type Gene Clusters

A comparative analysis revealed that the plasmids of different Inc groups (pJP4, plasmid5, pEMT1, and pkk4) shared a high synteny and a sequence identity region that included the *tfd*_I_, *tfd*_II_*SA* clusters, and the accessory genes. In addition, in all plasmids, except pkk4, this region also included *xerC/D* and transposase of Tn*5504* genes (Figure 2b). The presence of the *tfd*_II_*SA* cluster, in addition to the *tfd*_I_ cluster amongst pJP4, plasmid5, pEMT1, and pkk4, has been shown previously [12]. The synteny region of the four plasmids did not include the two genes of plasmid pEMT1, *lacZ* and *aphA1*, encoding beta-galactosidase and aminoglycoside 3′-phosphotransferase, which are involved in lactose metabolism and antibiotic kanamycin resistance, respectively. Also, only plasmids pEMT1 and pkk4 had a truncated copy of the Tn*5504* transposase gene in the same orientation as the others.

In addition, all the plasmids contained ORF31 and ORF32, with unknown functions. The deduced proteins of these ORFs have completely identical regions of 208 amino acids, in length.

## 3. Discussion

The results suggest the occurrence of specific patterns in the distribution of *tfd* clusters (*tfd*_I_ and *tfd*_II_). They also enable the proposition of an alternative theory on *tfd* cluster architecture formation of the canonical plasmid, pJP4.

### 3.1. Distribution of tfd_II_ Gene Clusters among IncP-1 Plasmids

**Figure 3:**
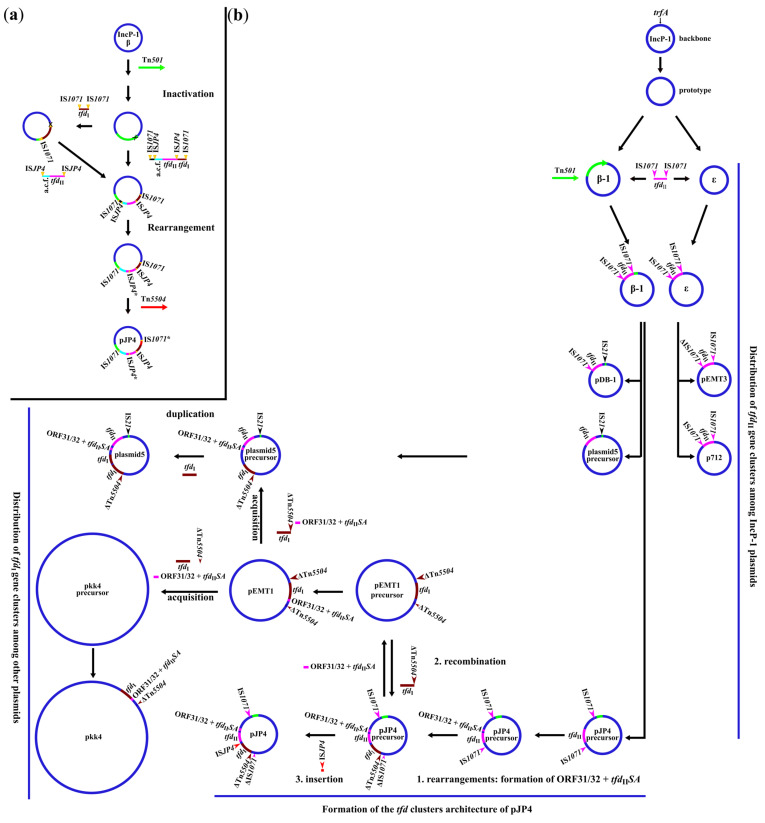
Putative distribution patterns of *tfd* clusters and the scenario of pJP4 plasmid architecture formation: (**a**) the previous model for the evolution of pJP4, adapted from Trefault et al. (2004) [3]; (**b**) the distribution patterns and scenario proposed in the present study. Plasmids are represented by circles, and the resulting evolutionary events by labeled arrows. Clusters and mobile elements acquired by plasmids are indicated by different colors on the plasmid circles. The truncated mobile elements are designated by the asterisk or delta symbol (Δ).

Recently, the *tfd* clusters of plasmids p712, pEMT3, pDB1, and plasmid5 were classified as complete *tfd*_II_ clusters possessing an eight-gene structure (*tfd*_II_*AKBFECDR*) [12]; in contrast to the seven-gene structure (*tfd*_II_*KBFECDR*) of the canonical plasmid, pJP4. At the same time, all plasmids carrying both complete and incomplete *tfd*_II_ clusters belonged to the β-1 and ε subgroups of the IncP-1 incompatibility group. The flanking of the *tfd*_II_ of plasmids p712 and pEMT3 from the ε subgroup by IS*1071* and direct nucleotide repeats indicates that *tfd*_II_ could be included in the composite transposon. This reveals a possible transposon-associated common initial distribution pattern of complete *tfd*_II_ clusters among IncP-1 plasmids. This is confirmed by the absence of *tfd*_I_ and *tfd*_II_ clusters among a number of plasmids, whose host bacteria were isolated from xenobiotic-contaminated soils (including 2,4-D) but belong to other incompatibility groups [15,16,17]. The lower identity of the *trfA* gene between the β-1 and ε subgroups, rather than between their *tfd*_II_ clusters, indicates that the composite *tfd*_II_-containing transposon was already inserted into the precursor plasmids after they diverged into the two subgroups of lncP-1. Thus, the distribution of the probable *tfd*_II_-containing transposon occurred in two ways. One of them included insertion into an ‘empty’ precursor plasmid of the ε subgroup, which possessed only backbone genes; and further distribution as a part of the plasmids of this group (p712 and pEMT3). Another way occurred via the insertion of a *tfd*_II_-containing transposon into a β-1 subgroup precursor plasmid containing a Tn*501*-like transposon. It is known that Tn*501*-like transposons linked with the *mer* operon are widespread among plasmids of β-1 [14]. Most likely, the acquisition of the *tfd*_II_-containing transposon, as well as the further insertions of IS*21*, truncated the Tn*501*-like transposon and the *mer* operon of pJP4, plasmid5, and pDB-1 precursors. All the above mentioned resulted in the rise of two plasmid lineages containing *tfd*_II_ clusters. The possible distribution pattern of *tfd*_II_ clusters is visualized in Figure 3b.

Apparently, the putative *tfd*_II_ -containing transposon is currently inactive. This is suggested by the absence of intermediate replicons (e.g., plasmids of other incompatibility groups or chromosomes) that could serve as a source for IncP-1 plasmids. Thus, the distribution of the *tfd*_II_ cluster is plasmid-mediated only. Furthermore, plasmids of the β-1 promote a wider distribution of *tfd*_II_, compared to the ε subgroup.

Among the plasmids of the β-1 subgroup, pDB-1 is the most ancient member with a *tfd*_II_ cluster, and the most related to the common plasmid precursor. Other members, namely, pJP4 and plasmid5, underwent further evolutionary changes.

### 3.2. Formation of the tfd Cluster Architecture of pJP4

The theory of pJP4 plasmid formation was proposed by Trefault and colleagues (2004). According to this theory, the plasmid precursor acquired the mercury resistance transposon Tn*501*, which contained the *mer* operon. It was then inactivated by an IS*1071*-based transposition linked with the *tfd*_I_ gene cluster. The precursor plasmid further acquired the *tfd*_II_ cluster via IS*JP4*-based transposition with the *tfd*_I_ cluster, either before or after its integration into the precursor. This was followed by the internal rearrangement and the inactivation of one IS*JP4* copy. Subsequently, one copy of IS*1071* was also further inactivated by Tn*5504*, which was inserted into the plasmid precursor (Figure 3a).

The above proposed a probable, common distribution pattern of *tfd*_II_ rather than *tfd*_I_ clusters within the composite IS*1071*-based transposon among the plasmids of the IncP-1 group. This, and other obtained findings, make it possible to propose another scheme of formation of the *tfd*_I_ and *tfd*_II_ clusters’ architecture of the pJP4 plasmid after the acquisition of *tfd*_II,_ as a part of the composite transposon.

The first significant step was the formation of the cluster of two ORFs, namely ORF31 and ORF32 (further ORF31/32), which resulted from the duplication of the *tfdR*_II_ gene and the sequence of ORF31 on the opposite strand of the pJP4 plasmid. This led to the formation of ORF31/32 and the *tfdS*_II_ (=*tfdR*_II_ gene copy). The subsequent transfer of the *tfdA*_II_ gene from a site immediately downstream of the *tfdK*_II_ gene to a site immediately upstream of *tfdS*_II._ resulted in the formation of the ORF31/32 and *tfd*_II_*SA* structures (Figure 3b). Previously, Trefault and colleagues (2004) assumed only a duplication of the *tfdR*_II_ gene.

The following second step was the recombination event between the precursors of pEMT1 and pJP4. As a result, the pEMT1 precursor plasmid acquired a region containing ORF31/32 with *tfd*_II_*SA* clusters and a number of accessory genes. Whereas the plasmid precursor pJP4 acquired a region containing the genes *xerC/D* and the transposase of Tn*5504*, as well as the *tfd*_I_ cluster (Figure 3b). This caused a truncation of the IS*1071*. Also, as indicated above, it was shown that the putative transposon, Tn*5504*, lacked the resolvase gene which is required for the transposition of Tn*3* family members. Thus, the assumption of Trefault and colleagues (2004) about the inactivation of one copy of IS*1071* by insertion of the Tn*5504*, is not confirmed.

At the third step, IS*JP4* was inserted into 3’ end of the *tfdT* gene of the *tfd*_I_ cluster of the pJP4 plasmid precursor, resulting in its truncation. Confirmation that this was the final step is provided by the fact that the complete sequence of the *tfdT* gene was observed in other plasmids (pEMT1, pkk4, and plasmid5). According to Nguyen and colleagues (2019), all plasmids of the IncP-1 type of the pPO line possess IS*JP4* analogous to the pJP4 plasmid (3’ end of the *tfdT*). However, although some of the *tfd*_II_ clusters do not contain the *tfdB*_II_ and *tfdR*_II_ genes, the occurrence of such canonical elements as the *tfd*_II_*SA* cluster and IS*JP4* indicates that these plasmids are variations of the canonical plasmid, pJP4. Interestingly, only plasmids of the pPO line and pJP4 possess a complete IS*JP4*, in contrast to the other plasmids analyzed. This also supports the initial IS*1071*-based distribution of *tfd*_II_ instead of the IS*JP4*-based distribution according to the previous theory.

In summary, the *tfd*_I_ and *tfd*_II_ clusters’ architecture of the canonical plasmid, pJP4, was formed via the sequential processes of internal rearrangements, recombination, and IS*JP4* insertion.

### 3.3. Distribution of tfd_I_ Gene Clusters

Only plasmids of the IncP-1 type are carriers of *tfd*_II_ clusters and a combination of *tfd*_I_ and *tfd*_II_ clusters. The absence of carriers of only *tfd*_I_ clusters among them, as well as the belonging of *tfd*_I_ and *tfd*_II_ to different evolutionary lineages [12], indicates that plasmids of other Inc groups initially provide the main contributions to the distribution of *tfd*_I_. In addition to the above-mentioned, IncHI2 and IncA/C (plasmids pEMT1 and pkk4, respectively), it is probable that plasmids from other groups may also be involved. This is supported by the identification of *tfd*_I_ and *tfd*_II_*SA* clusters in the contigs of the unidentified pPO4 plasmid of the pPO line [11].

Based on the results on synteny between plasmids pJP4, plasmid5, pEMT1, and pkk4, the following scenario can be hypothesized (Figure 3b). The pEMT1 precursor acquired a region containing ORF31/32, the *tfd*_II_*SA* cluster, and a number of accessory genes as a result of recombination, as described in the second step of the formation of the pJP4 plasmid architecture. Furthermore, plasmid5 and pkk4 acquired a region containing the *tfd*_I_, ORF31/32, the *tfd*_II_*SA* cluster, and a number of accessory genes. In addition, plasmid5 acquired a region containing the genes *xerC/D*, and the transposase of Tn*5504* flanking the *tfd*_I_ cluster from the 3′ end of the *tfdB*_I_ gene (similar to pJP4). And plasmid pkk4 acquired another more truncated transposase gene of Tn*5504* flanking the accessory gene region (Figure 2b). Thus, plasmid pEMT1 could serve as an intermediate replicon between plasmids pJP4 and plasmid5 belonging to the same Inc group. A partial duplication of the *tfd*_I_ cluster in the precursor of plasmid5 led to the final formation of the plasmid5 structure. An interesting observation: the higher identity between the ORF31/32 and *tfd*_II_*SA* clusters of pJP4 plasmids and plasmid5, compared to the *tfd*_II_ clusters of the same plasmids, confirms that ORF31/32 and *tfd*_II_*SA* were acquired by plasmid5 much later than the time when *tfd*_II_ was obtained by the common ancestor through the IS*1071*-based transposition.

As a result of the above, a pattern of predominant distributions of *tfd*_I_ and *tfd*_II_ clusters by IncP-1 plasmids of the β-1 subgroup was formed, in addition to the pattern of the initial distribution of *tfd*_I_ clusters by non-IncP-1 plasmids.

It is important to mention that the order of events in the distribution of *tfd* clusters and the formation of the pJP4 architecture may differ from that proposed above. Since the proposed scheme in Figure 3b is based on already-sequenced plasmids, the immediate participants could possibly already be eliminated or not yet sequenced. Nevertheless, an approach based on comparative genomics and a subsequent analysis of possible evolutionary events can provide new insights into both the evolutionary biology of plasmids and their epidemiology. For example, Moran and colleagues (2024) [18] traced the evolutionary lineage of pQEB1-like plasmids encoding the carbapenemase *bla*_KPC-2_ gene, which were responsible for a hospital outbreak of clinical infections. And among plasmids of the IncX3 incompatibility group in China, high conservation of the backbone region was shown to remain for over 10 years. At the same time, the genetic context of *bla*_NDM_ resistance was falling into five subtypes [19]. Also, new insights into the evolution of L and M plasmid lineages and their division into five and eight types, respectively, were revealed [20].

In summary, it is obvious that pJP4-like plasmids (pJP4, plasmid5, and pPO line plasmids) are key mediators of the distribution of the *tfd*_I_ and *tfd*_II_ clusters among bacterial populations. Interestingly, the ORF31/32 and *tfd*_II_*SA* structures originating from pJP4 in all four plasmids serves as a kind of fingerprint. The discovery of plasmid5 with a complete two cluster *tfd* architecture and plasmids of the pPO line revealed that there is a whole lineage of pJP4-like plasmids with interesting evolutionary histories. Unfortunately, the absence of complete sequences of pPO line plasmids does not allow for a determination of whether their structure is identical to that of the canonical plasmid, pJP4, (despite deletions of some genes of the *tfd*_II_ cluster). Nevertheless, this intriguing fact offers hope for the existence of other pJP4-like plasmids with interesting features of *tfd* cluster architectures, and sheds light on the turbulent lifestyle amongst these plasmids. Regardless of the above, the application of strains with complete *tfd* gene clusters is more efficient from the point of view of environmental remediation strategies. In particular*, Cupriavidus oxalaticus* X32 with plasmid5 is currently more efficient in environmental remediation than the model degrader *Cupriavidus necator* JMP134 with a canonical pJP4 plasmid [10]. Apparently, similar bacterial strains with complete *tfd* clusters will dominate in the future.

## 4. Materials and Methods

### 4.1. Databases and Data Collection

All plasmid nucleotide sequences for the in silico analysis were obtained from the NCBI’s database records [21]. Insertion elements (ISs) were analyzed using blastp searches against the ISfinder database and repository, applying default parameters [22]. The Plasmid PubMLST-curated database [23] was exploited for typing IncA/C and IncHI2 plasmid types based on their incompatibility groups with default settings.

### 4.2. ORF Prediction and Annotation Sequence Definition

The prediction of open reading frames (ORFs) was provided by the web version of the NCBI ORF finder (https://www.ncbi.nlm.nih.gov/orffinder/ (accessed on 5 June 2023)). The UniProtKB/Swiss-Prot (swissprot) database was then used for NCBI blastp [21] similarity searches at default parameters to annotate predicted ORFs.

### 4.3. Comparative Genomic Analyses

The multiple alignment of nucleotide and protein sequences for subsequent inferring of phylogenetic trees and analyses of identity and similarity values were performed using the MAFFT algorithm of the https://www.ebi.ac.uk/Tools/msa/mafft/online (accessed on 16 July 2023) tool at default parameter settings [24]. SIAS (http://imed.med.ucm.es/Tools/sias.html (accessed on 16 July 2023)) was used to calculate the values of identity and similarity between the matching nucleotide and protein sequences of the explored plasmids. In order to illustrate IncP-1 plasmids synteny and *tfd* gene cluster rearrangements, a synteny analysis was carried out between both linearized and oriented plasmids or plasmid regions. Using Easyfig v. 2.2.5 [25], detailed synteny maps were visualized, and blastn identity comparisons at default settings were performed.

### 4.4. Phylogenetic Analyses

The evolutionary analyses were conducted in MEGA7 software v. 7.0.26 with 1000 bootstrap replicates [26] using the Maximum Likelihood method based on the JTT matrix-based model.

## 5. Conclusions

Here, based on the results of phylogenetic and comparative genomic analysis, patterns of *tfd* clusters distribution are revealed, and a new theory of *tfd* clusters architecture formation of pJP4 plasmids is proposed. Despite the findings clearly indicating the existence of a whole lineage of pJP4-like plasmids, pJP4 plasmids continue to serve as key mediators of plasmid rearrangements associated with *tfd* clusters. Future exploration will definitely expand the history of this amazing plasmid and highlight new trajectories in the evolution of IncP-1 plasmids. Quite ironically, the first sequenced canonical 2,4-D degradation plasmid has both *tfd* clusters in truncated form. Obviously, bacterial strains with full *tfd* clusters should be used for more efficient environmental bioremediation.

## Figures and Tables

**Table 1:** List of the plasmids harboring *tfd* clusters.

Plasmid	Replicon Type (Inc Group)	Size, bp	Host Strain	Structure of *tfd* Type Cluster	GenBank Accession No.	Reference
pJP4	IncP-1 (β-1)	87,688	*Cupriavidus pinatubonensis* JMP134	*tfd*_I_*BFEDCT*, *tfd*_II_*SA*, *tfd*_II_*KBFECDR*	AY365053	[3]
plasmid5	IncP-1 (β-1)	89,459	*Cupriavidus oxalaticus* X32	*tfd*_I_ *BFEDCT, tfd*_I_ *BFED, tfd*_II_ *SA, tfd*_II_ *AKBFECDR*	CP038640	[10]
pDB1	IncP-1 (β-1)	65,269	*Variovorax* sp. DB1	*tfd*_II_ *AKBFECDR*	JQ436721	[8]
p712	IncP-1 (ε)	62,798	*Ralstonia pickettii* 712	*tfd*_II_ *AKBFECDR*	JQ436722	
pEMT3	IncP-1 (ε)	63,472	Uncultured bacterium	*tfd*_II_ *AKBFECDR*	JX469827	[9]
pEMT1	IncHI2	100,133	*Paraburkholderia hospita* DSM 17164	*tfd*_I_*BFEDCT*, *tfd*_II_*SA*	CP026110	[13]
pkk4	IncA/C	568,203	*Burkholderia* sp. KK1	*tfd*_I_*BFEDCT*, *tfd*_II_*SA*	CP016005	unpublished

## Data Availability

Data is contained within the article.
